# Macrolide Therapy in Chronic Inflammatory Diseases

**DOI:** 10.1155/2012/636157

**Published:** 2012-08-21

**Authors:** Brygida Kwiatkowska, Maria Maślińska

**Affiliations:** Department of Early Diagnosis of Arthritis, Institute of Rheumatology, Spartanska 1, 02-627 Warsaw, Poland

## Abstract

Macrolides are a group of antibiotics with a distinctive macrocyclic lactone ring combined with sugars (cladinose, desosamine). The action of macrolides is to block protein synthesis by binding to the subunit of 50S ribosome of bacteria. Prototype macrolide was erythromycin, which came into clinical practice in the 50s of the 20th century. Its antimicrobial spectrum covers the scope of the penicillins but is extended to the impact of atypical bacteria. In the 90s more drugs of this group were synthesized—they have less severe side effects than erythromycin, extended spectrum of Gram-negative bacteria. Macrolides are effective in treating mycobacterial infections especially in patients infected with HIV. It is now known that in addition to antibacterial abilities, macrolides have immunomodulatory effects—they inhibit the production of proinflammatory cytokines (TNF, IL1, 6, and 8) affect transcription factors (NF-**κ**B) as well as costimulaton (CD 80) and adhesion molecules (ICAM). This review article focused not only on the their antimicrobial abilities but also on efficacy in the treatment of several inflammatory disorders independent of the infectious agent. Their wider use as immunomodulators requires further study, which can lead to an extension of indications for their administration.

## 1. Introduction

The name “macrolide” covers a family of different antibiotics produced by fungi of the genus *Streptomyces* and some bacteria such as *Arthrobacter* spp. Construction of macrolides is based on the large macrocyclic lacton ring, the activity of which is due to the presence of macrolide ring containing one or more deoxy sugar (usually cladinose-neutral sugar and desosamine-amino sugar). Lactone rings usually consist of 14, 15 or 16 members.

**Figure 1:**
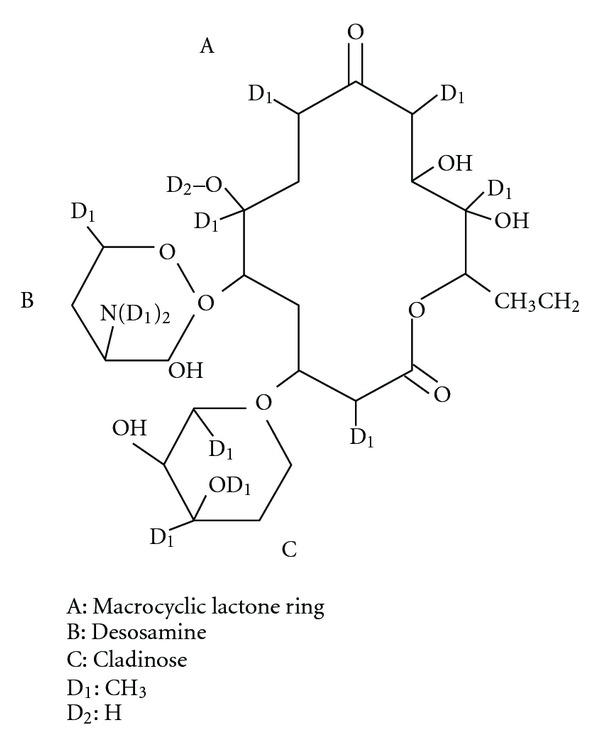
14 member lactone rings of erythromycin.

Erythromycin is a macrolide prototype—it contains 14-membered lactone rings, (Figure 1). Its first clinical use in the upper respiratory tract infections occurred in the 50s of the 20th century. Other macrolides with 14-membered ring include clarithromycin, dirithromycin, oleandomycin, roxithromycin, and 16-membered ring: josamycin, midecamycin, mikamycin, and spiramycin. Also stands out azalide—15-membered ring macrolide—azithromycin, and, we can also distinguish ketolides with 14-membered ring such as telithromycin and cethromycin. Tacrolimus isolated from *Streptomyces tsukubaensis* and sirolimus isolated from *Streptomyces hygroscopicus* also belong to this group of antibiotics (Figure 3). 

## 2. The Mechanism of Antibacterial Action of Macrolides

Macrolide antibiotics have been used for many years to treat infectious diseases. Macrolides antibacterial mechanism of action involves binding to the 50S ribosomal subunit, which causes inhibition of the biosynthesis on ribosomal protein level [1, 2]. Both macrolides and ketolides bind domain V of 23S ribosomal RNA (rRNA), contained in the 50S subunit of bacterial ribosomes. However, ketolides have from 10 to 100 greater affinity for the ribosome than erythromycin. Ketolides also, unlike the macrolides, have a greater affinity for binding to the 23S rRNA domain II, which allows them to maintain activity against bacterial strains that are resistant to macrolides due to changes in domain V of 23S [3].

**The Spectrum of Antibacterial Activity**

Macrolides have become an alternative for people allergic to penicillin. The first macrolide erythromycin included in its scope spectrum like penicillins, but also demonstrated the effectiveness of intracellular microorganisms such as *Legionella pneumophila, Chlamydia* spp,and *Mycoplasma.* Further discovery and subsequent synthesis of macrolides increased their scope of activity of *Helicobacter pylori* and *Mycobacterium.* The scope of macrolides effect includes also *Bacillus anthracis, Bordetella reccurentis, Corynebacterium diphtheriae*, *Listeria monocytogenes*, *Streptococci* (*S. pneumoniae*), and methicillin-sensitive *Staphilococcus*. They act also on the *Treponema pallidum*, *Toxoplasma gondii*, *Plasmodium* spp, and *Cryptosporidium* [4].

## 3. Immunoregulation and Anti-Inflammatory Action of Macrolides

In recent years, it has been shown that macrolides beyond the bacteriostatic and bactericidal effect have also anti-inflammatory effect, which was used in chronic inflammatory diseases such as atopic dermatitis, nonspecific inflammatory bowel disease, psoriasis, and arthritis. The effect of macrolides on the inflammatory cell activity by influencing the production and release of proinflammatory cytokines has been demonstrated in many studies. Cytokines and chemokines play a key role in regulating both the proinflammatory immune response—tumour necrosis factor (TNF-), granulocyte—macrophage colony-stimulating factor (GM-CSF), interleukin-L IL-1, IL-6, IL-8, and interferon gamma (IFN-) and anti-inflammatory (e.g., IL-10). 

It was shown that macrolides inhibit the production and secretion of IL-1SS and TNF-. in monocytes [5] and IL-1SS, IL-6, TNF-., and GM-CSF in mast cells [6], and IL-8 protein epithelial neutrophil-activating (ENA-78) macrophage inflammatory protein (MIP-1) in macrophages and leukocytes [7]. It was also shown that clarithromycin suppresses the production of IL-6 and IL-1SS by fibroblast-like cells of the synovial membrane [8]. Therapeutic concentrations of erythromycin and clarithromycin reduce the expression of IL-8 mRNA level in bronchial epithelial cells of patients with chronic inflammatory airway disease [9].

Erythromycin also affects the neutrophils migration [10], proliferation of lymphocytes [11], and differentiation of monocytes [12]. Expression of genes involved in immune response and inflammation (e.g., iNOS, COX-2, TNF-alpha, IL-1, and IL-6) at the level of transcription is regulated by nuclear factor-kappa B (NF-*κ*B) [13]. Erythromycin and roxithromycin exhibit antioxidant properties and prevent activation of (NF-*κ*B) [14]. 

Erythromycin and clarithromycin also show a concentration-dependent inhibition of IL-8 release by eosinophils isolated from people with atopic dermatitis [15]. Macrolides inhibit as well the secretion of eosinophilic chemotactins, cytokines RANTES, and eotaxin in lung fibroblasts [16]. It was also found that macrolides may alter the ratio of IFN-./IL-4 (Th1/Th2) [17]. Macrolides also affect dendritic cells (from mouse bone marrow) by the increase in the expression of CD80, a molecule co-stimulatory T-cell activation [18]. Azithromycin causes increased production of IL-10, while clarithromycin inhibits the production of IL-6 by dendritic cells. All these studies show different effects of macrolides on cytokine production and release of pro- and anti-inflammatory cytokines. Such effects apply only to 14- and 15-membered macrolides [19].

**Impact on Other Immunomodulating Mechanisms**

Macrolides may influence the metabolism of arachidonic acid by lipoxygenase—modulation cycle of lipoxygenase modulation. Erythromycin and roxitromycin reduce the number and activity of chemotactic neutrophills through the reduction of leukotriene B4 (LBT4) [20].

Several recent studies show the impact of macrolides on the phenomenon of apoptotic epithelial cells and macrophages [21, 22]. In addition, they inhibit angiogenesis by inhibiting the production of vascular endothelial growth factor (VEGF) stimulated by TNF-alpha [23]. The effect of macrolides on the transduction pathways of many different external signals MAPK (mitogen-activated protein kinase) is not limited to the production of cytokines. Erythromycin inhibits IL-1 inducing phosphorylation of p38 MAPK in rheumatoid synovial cells in vitro [24]. Inflammatory cells can produce isoforms of NO using the induced synthesis of nitric oxide (iNOS), which increases the inflammation and causes the destruction of cells. It has been shown in vitro that the macrolides inhibit the production of NO [25, 26]. 

## 4. Clinical Practice—Macrolides Use

### 4.1. Airway Diseases

**Figure 2:**
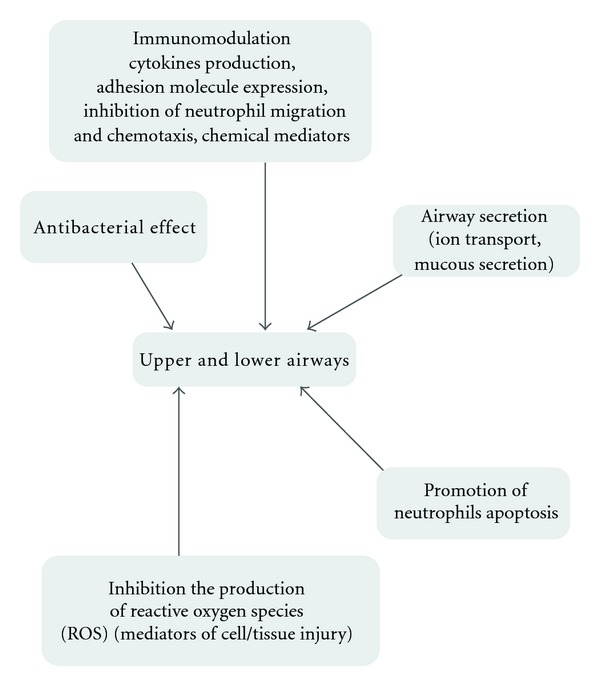
Macrolides influence on inflammatory airway diseases.

**Figure 3:**
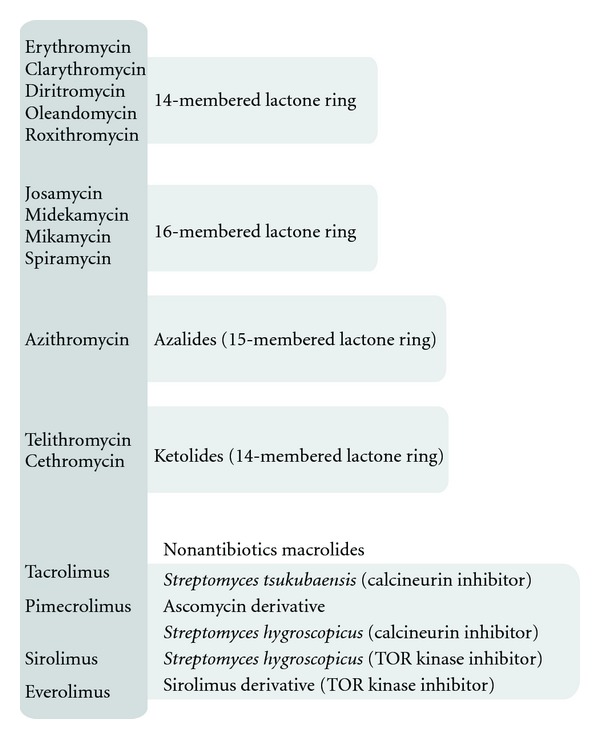
Selected macrolides.

**Figure 4:**
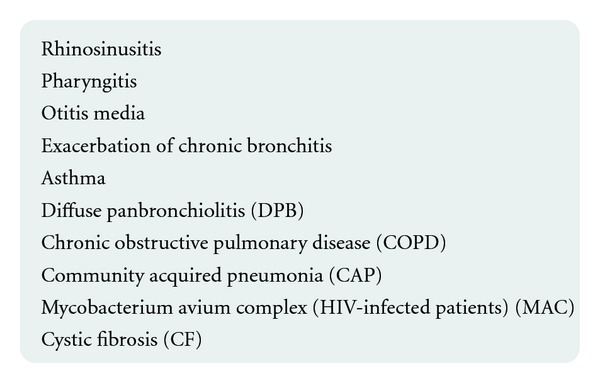
Airway diseases in which macrolides are indicated.

The most widely from beginning of the introduction into clinical practice, macrolides are used in the treatment of airway diseases. Because of their antibacterial and immunomodulatory abilities, a good tissue penetration and capability for intracellular action are of great importance as well as wide-broad efficacy against many organisms affecting lungs. It was demonstrated that in patients suffering from steroid-dependentasthma the concomitant use of the clarithromycin caused (through theinfluence of cytochrome P450 function) the increase in GKS concentrations, allowing for steroid dose reduction [27, 28]. Until now there is no sufficient evidence and recommendation to treat asthma, by macrolides for long-term therapy, however, it is obvious that atypical bacterial infection in asthma patients is the indication for macrolides therapy [27] (Figures 2 and 4). Several other macrolide properties, such as anti-inflammatory action and production of cytokines (e.g., IL8-a neutrophil chemoattractant), influence on neutrophil migration, antibacterial effect on colonization, and infection by *Pseudomonas aeruginosa*, *Chlamydia pneumonia*, and Mycoplasma pneumonie, may prove beneficial in other various airway diseases. These include diffuse panbronchiolitis (DPB) [29, 30], chronic obstructive lung disease, cystic fibrosis (CF), and bronchiolitis obliterans syndrome (BOS), the latter occurring as a lung transplant complication [31].

### 4.2. The Use of Macrolides in the Treatment of Skin Diseases

Immunosuppressive macrolides are a new class of anti-inflammatory substances used in the treatment of skin diseases. Tacrolimus (FK506) and pimecrolimus when applied topically penetrate the skin and act locally immunoregulatory [32]. 

Pimecrolimus and tacrolimus are associated in the cytoplasm of target cells with a specific receptor protein called macrophyllin-12, known as tacrolimus binding protein FKBP (FK506-binding protein). Tacrolimus/pimecrolimus-macrophyllin-12 blocks calcineurin complex. The inhibition of calcineurin results in a lack of gene expression of many mediators of inflammation [33, 34]. 

Tacrolimus has immunosuppressive activity similar to cyclosporine A, pimecrolimus has a stronger effect. Both drugs were used in the treatment of atopic dermatitis (AD), psoriasis, and contact dermatitis. 

Sirolimus (rapamycin) is also a macrolide, but with a different site of action than tacrolimus and pimecrolimus. In the complex with the cytosolic protein FKBP-12, it causes the inhibition of TOR (target of rapamycin) and thereby inhibits intracellular signals pathway conduction. Sirolimus, that acts on T cells, has an effect on angiogenesis by reducing the production of vascular endothelial growth factor (VEGF). Sirolimus was used in the treatment of psoriasis. The advantage of the use of macrolides for the treatment of skin diseases, both locally and topically, is that they have no effect on collagen synthesis and thus they do not cause skin atrophy in contrast to the glucocorticoids. 

Clinical studies have confirmed the effectiveness of oral therapy with macrolide group antibiotics of psoriasis vulgaris [35]. It was shown that 4 weeks of treatment of patients with skin psoriasis with oral macrolides combined with topical treatment with corticosteroids significantly reduced the Psoriasis Area and Severity Index (PASI) and has an impact on the abolition of itching [36]. 

### 4.3. Macrolides in Treatment of Nonspecific Inflammatory Bowel Diseases

Due to the immunomodulating effect of macrolides, antibiotics are increasingly used in nonspecific inflammatory bowel diseases, especially Crohn's disease. 2-year observation of patients with Crohn's disease treated with following combination therapy: rifabutin with a macrolide (azithromycin or clarithromycin) for a period of 6 to 35 months showed significant improvement in the assessment of disease activity (Harvey-Bradshaw Crohn's activity index) effect after 6 months of therapy and continuing for the next 24 months [37]. Another study using clarithromycin as immunomodulating drug for 24 weeks and longer showed that 42.9% of patients with active Crohn's disease had remission in the assessment of CDAI (Crohn's Disease Activity Index) after 12 weeks of treatment [38].

**Eradication of H. pylori—a Permanent Place for the Use of Macrolides**

A lot of studies demonstrate the effectiveness of clarithromycin in the eradication of *H. pylori* infection in combination with another antibiotic and antisecretory agent (proton pump inhibitor-PPI) as standard triple therapy. However, the increasing resistance to the clarithromycin can be observed [39].

## 5. Prokinetic Effect of Macrolides

It has been demonstrated that 14-membered lacton ring macrolides stimulate gastrointestinal motility, while there is no such effect of the 15- and 16-membered lactone ring macrolides use. It is known that erythromycin acts on the intestinal and gallbladder motility through motilin receptor which causes stimulation of enteric nerves and smooth muscle [40, 41]. Erythromyicin activity, in particular on gastric antral motility, has been also demonstrated to be mediated via cholinergic pathway and activation of a neuromuscular receptor [41]. The attention paid to the prokinetic properties of macrolides is associated with the ongoing search for the effective treatment of gastrointestinal disorders such as gastroparesis in diabetic patients, slow emptying and gastroparesis in intensive care patients undergoing mechanical ventilation, and gastroesophageal reflux and bacterial overgrowth in intensive care patients during enteral nutrition. The prokinetic qualities of macrolides may also be considered in the use of these antibiotics in lung transplant patients, where the risk of graft dysfunction is increased by gastroesophageal reflux (GERD). It is suggested in the literature that erythromycin prokinetic efficacy is dependent on the dose, as it decreases in the days following application. The use of macrolides is associated with risk of inducing and increasing bacterial resistance to macrolides and other side effects, such as arrythmias with prolonged QT interval (ventricular tachycardia—“torsades de pointes”). There is no strong recommendation for macrolide use as a first-line prokinetic treatment. We should consider their use in cases of failure of all other gastrointestinal hyipomotility treatments (e.g., metoclopramide) and of complications of gastrointestinal motility disorders [42, 43]. 

## 6. The Use of Macrolides in Rheumatoid Arthritis and Other Rheumatic Diseases

The immunosuppressive effect of tacrolimus is well known in patients with rheumatoid arthritis (RA) for whom methotrexate was ineffective [44] as well as the immunosuppressive effects of sirolimus on the growth of synovial fibroblasts in patients with rheumatoid arthritis [45]. The results of a recent study have demonstrated the effectiveness of roxitromycin as disease-modifying drug in the early forms of rheumatoid arthritis [46]. Clarithromycin showed similar efficacy [47], but it is not a standard therapeutic procedure in the treatment of RA. The application of roxitromycin both in early and late periods of rheumatoid arthritis can be an effective form of therapy that modifies the course of the disease, but requires further studies [48]. In this paper, Matsuoka et al. demonstrated the inhibitory effect of erythromycin costimulating molecule and production of proinflammatory cytokines by synovial fibroblast-like cell. The authors suggested the possibility of further studies in patients with RA [8].

**Reactive Arthritis (ReA)**

In this group of patients particularly chlamydia-induced ReA is an indication for antibiotic therapy. Good effects of treatment Ch– and ReA are described in the application of tetracycline, ciprofloxacin, and doxycycline with rifampicin [49]. Greater efficiency was obtained when using azithromycin and rifampicin [50]—this treatment is particularly effective in the treatment of *Chlamydia pneumoniae* infection [51].

### 6.1. Conjunctivitis

The studies have shown that the use of azithromycin in the form of eye drops for bacterial conjunctivitis can remove most microorganisms that can cause the inflammation [52]. 

### 6.2. Trachoma

Chronic inflammation of the cornea and conjunctiva caused by serotypes A, B, Ba, and C Chlamydia trachomatis, which is the most common cause of blindness in developing countries. In the case of the disease, the drug of choice is azithromycin administered orally (single dose efficacy adults 1 g, children 20 mg per kg) and topical tetracycline [53]. 

### 6.3. The Effect of Macrolides on Viral Upper Respiratory Tract

The studies in recent years have shown that macrolides can inhibit the development of viral infection of upper respiratory tract. Clarithromycin, by inhibiting the production of intracellular adhesion molecule ICAM-1 and secretion of IL-6 and IL-8, significantly influences the pathophysiological changes associated with infection caused by rhinovirus (RV). Clarithromycin inhibits protein and mRNA expression of ICAM induced by infection with the virus and increased levels of proinflammatory cytokines such as IL-1SS, IL-6, and IL-8. This effect is the greatest 3 days after the infection [54] and similar to the effects demonstrated by erythromycin [55]. Similar effects were demonstrated in the case of azithromycin and paramyxovirus infections, particularly respiratory syncytia virus (RSV) [56] and clarithromycin and its effect on infection with influenza virus type A [57]. Macrolides may have future use in the inhibition of chronic inflammation induced by upper respiratory viral infections, such as RV, RSV, or influenza A. 

## 7. New Possibilities of Macrolides

Drugs to build a macrolide such as sirolimus or its derivative everolimus both inhibit the TOR kinases and the proliferation and clonal expansion, therefore, they were applied in transplant rejection reactions as well as in interventional cardiology for coating stents (drug eluting stents), which lowers the risk of restenosis [58]. Further studies are underway on the macrolides, in which no evidence of antibacterial activity was found—only immunomodulating/immunoregulating functions. One of these is a macrolide CSY0073—azithromycin structure showing immumoregulating action in experimental models of inflammatory bowel disease and arthritis [59]. 

In recent years, it was also revealed that the impact of rapamycin on the inhibition of cell aging which can be important in treating progeria and other age-related diseases [60].

## 8. Antibacterial Action and Resistance Mechanisms for Macrolides-Clinical Problem 

Antibiotic resistance can be the result of adenine methylation associated with the domain V of 23S rRNA, which causes the insensitivity of such a ribosome to macrolides [3]. The resistance to esterase production may also occur. This enzyme, which hydrolyses macrolide, is produced by Enterobacteriaceae. The cause of resistance of bacteria (mainly G) can constitute negative disturbances and abnormal permeability of outer membrane flow hydrophobic molecules.

Cross-resistance to erythromycin and other macrolides can occur as well as cross-resistance to macrolides and clindamycin and streptogramin B—which bind to the same place on the ribosome.

## 9. Interaction of Macrolides with Other Drugs and the Resulting Toxicity of Drugs

Macrolides inhibit the activity of cytochrome P-450 and its isoform as CYP 3A4 [61]. Macrolides can be divided into 3 groups according to the inhibition of CYP 3A4. Erythromycin and troleandomycin are the strongest inhibitors of cytochrome CYP 3A4. Clarithromycin shows weak inhibition of CYP 3A4, whereas in vitro studies of azithromycin and diritromycyna show almost no inhibition of the cytochrome [62]. Inhibition of CYP3A4 changed metabolism of many drugs, increasing their concentration in serum and exceeding therapeutic levels and thus is the cause of their toxic effects. Special attention should be paid to the potential toxic effects of benzodiazepines, oral anticoagulants (warfarin), theophylline, neuroleptics, statins, and class IA antiarrhythmic drugs such as quinidine and digoxin toxicity risk [63, 64]. Macrolide drugs may also prolong the QT interval and cause torsade pointes. 

The most common side effects of this drug class are disorders of the gastrointestinal tract (vomiting, diarrhoea, increased peristalsis). Allergic reactions with eosinophilia, pruritic skin, and urticaria are less common but also observed. In the course of their use, vasculitis (after i.v. administration), elevated transaminases, and hepatitis with cholestasis may occur. 

## 10. Conclusion

Since the discovery of erythromycin and its clinical use as an alternative to penicillin for the introduction of new macrolides such as azithromycin, clarithromycin, telithromycin, which are characterized by greater bioavailability, longer half-life, and extended-antibacterial spectrum and less severe adverse reactions, new abilities of macrolides were discovered. A new class of drugs that have no antibacterial abilities and have been applied not only to treat bacterial infections caused by common G+ bacteria and to a lesser extent G− but also demonstrated their effectiveness in treating atypical infections with bacteria, some protozoa (e.g., *T*. *Gondii*, *Leishzmania donovani*). They are used in mycobacterial infection (*Mycobacterium avium*). It has been shown that their antibacterial effectiveness involves not only the direct effect on the inhibition of bacterial protein biosynthesis but also their effects on the immune system. Thanks to the influence of co-stimulating particles (CD 80), proinflammatory cytokines production (TNFa, IL1, 6, and 8) and anti-inflammatory cytokines (IL-10), adhesion proteins (ICAM 1), the influence on intracellular signalling pathways, and functions of T cells, their wider use is possible in the treatment of inflammatory conditions beyond the control of infection. Further studies aim to find new indications for macrolides already used in clinical practice and to invent new macrolides of the main immunomodulating action.
